# Supplementary material for: The Systems Biology Research Tool: evolvable open-source software
Source: BMC Syst Biol. 2008 Jun 29;2:55. doi: 10.1186/1752-0509-2-55 (PMC2446383; doi:10.1186/1752-0509-2-55)
Supplement: Additional file 1 — SBRT Archive. An archive of the current version of the Systems Biology Research Tool. [file 1752-0509-2-55-S1.zip › sbrt-1.4.0/doc/developers_guide/api/sbrt/kernel/utilities/proc/VariableParticipation.html]

VariableParticipation


|  |  |  |
| --- | --- | --- |
| **Overview** \| **Package** \| **Class** \| **Use** \| **Tree** \| **Deprecated** \| **Index** \| **Help** | |  |
| **PREV CLASS**   **NEXT CLASS** | **FRAMES**    **NO FRAMES**     **All Classes** |  |
| SUMMARY: NESTED \| FIELD \| CONSTR \| METHOD | DETAIL: FIELD \| CONSTR \| METHOD |  |


---


## sbrt.kernel.utilities.proc Class VariableParticipation<E extends MathExpr<V>,V>

```
java.lang.Object
  sbrt.kernel.utilities.proc.VariableParticipation<E,V>
```

**Type Parameters:**: `E` - the expression type.: `V` - the variable type.

**All Implemented Interfaces:**: KernelProcess<java.util.Map<V,java.util.List<E>>>

---

``` public class VariableParticipation<E extends MathExpr<V>,V> extends java.lang.Object implements KernelProcess<java.util.Map<V,java.util.List<E>>> ```

This class is used to organize a collection of mathematical expressions
according to the variables they contain.

**Author:**
:   This class was written and documented by Jeremiah Wright
    while in the Wagner lab.

---

**Constructor Summary**

`VariableParticipation()`


| **Method Summary** | |
| --- | --- |
| `void` | `run(ResultsManager<java.util.Map<V,java.util.List<E>>> resultsManager)`             Adds to the provided results manager a map of variables mapped to a list of mathematical expressions in which they participate. |
| `void` | `setExprs(java.util.Collection<? extends E> expressions)`             Sets the collection of mathematical expressions to organize. |

**Methods inherited from class java.lang.Object**

`clone, equals, finalize, getClass, hashCode, notify, notifyAll, toString, wait, wait, wait`

**Constructor Detail**

### VariableParticipation

```
public VariableParticipation()
```


**Method Detail**

### setExprs

```
public void setExprs(java.util.Collection<? extends E> expressions)
```

:   Sets the collection of mathematical expressions to organize.

    :   **Parameters:**: `expressions` - the collection of mathematical expressions to organize.

---


### run

```
public void run(ResultsManager<java.util.Map<V,java.util.List<E>>> resultsManager)
```

:   Adds to the provided results manager a map of variables mapped to a
    list of mathematical expressions in which they participate.

    :   **Specified by:**: `run` in interface `KernelProcess<java.util.Map<V,java.util.List<E extends MathExpr<V>>>>`
    :   **Parameters:**: `resultsManager` - the results manager to which the map is added.


---


|  |  |  |
| --- | --- | --- |
| **Overview** \| **Package** \| **Class** \| **Use** \| **Tree** \| **Deprecated** \| **Index** \| **Help** | |  |
| **PREV CLASS**   **NEXT CLASS** | **FRAMES**    **NO FRAMES**     **All Classes** |  |
| SUMMARY: NESTED \| FIELD \| CONSTR \| METHOD | DETAIL: FIELD \| CONSTR \| METHOD |  |


---
